# The Deception of Certainty: how Non-Interpretable Machine Learning Outcomes Challenge the Epistemic Authority of Physicians. A deliberative-relational Approach

**DOI:** 10.1007/s11019-022-10076-1

**Published:** 2022-05-10

**Authors:** Florian Funer

**Affiliations:** Eberhard Karls University, Tübingen, Germany

**Keywords:** Machine learning (ML), Epistemic authority, Accuracy, Interpretability, Deliberation, Physician-patient relationship, Trust

## Abstract

Developments in Machine Learning (ML) have attracted attention in a wide range of healthcare fields to improve medical practice and the benefit of patients. Particularly, this should be achieved by providing more or less automated decision recommendations to the treating physician. However, some hopes placed in ML for healthcare seem to be disappointed, at least in part, by a lack of transparency or traceability. Skepticism exists primarily in the fact that the physician, as the person responsible for diagnosis, therapy, and care, has no or insufficient insight into how such recommendations are reached. The following paper aims to make understandable the specificity of the deliberative model of a physician-patient relationship that has been achieved over decades. By outlining the (social-)epistemic and inherently normative relationship between physicians and patients, I want to show how this relationship might be altered by non-traceable ML recommendations. With respect to some healthcare decisions, such changes in deliberative practice may create normatively far-reaching challenges. Therefore, in the future, a differentiation of decision-making situations in healthcare with respect to the necessary depth of insight into the process of outcome generation seems essential.

## Introduction

As firm steps of the digital transformation in healthcare, the focus in recent years has increasingly been on support systems whose mode of functioning is based on forms of ‘Machine Learning’ (ML). Connected with increasingly powerful processor technologies, such ML systems are already enabling improvements in accuracy and efficiency across several medical fields (Topol 2019; Esteva et al. 2019). Whereas such ML systems have so far helped to identify and evaluate potential differential diagnoses, mainly in the context of imaging procedures such as radiology (Hosny et al. 2018), dermatology (Patel et al. 2021), ophthalmology (Kapoor et al. 2019), or pathology (Chang et al. 2019), more and more possibilities to determine ‘best’ therapy options are coming to the forefront. The list of expectations and hopes regarding the potentials of ML in healthcare is long (cf. Esteva et al. 2019; Ahuja 2019). Medical practice could become more accurate, more individually fitting, with fewer harm and side effects, less costly in the long term by preempting diseases and in this respect more preventive. Algorithms could liberate medical practice from human psychological errors of perception and judgment (cf. Neighbour 2016: 182–185). Finally, some researcher expressed the hope that doctors could spend the freed-up time with their patients (Topol 2019). However, to ensure a high quality of medicine and healthcare in the future, the expectation of such technical progress here cannot avoid a serious discussion of the connected moral challenges, with their respective epistemic presuppositions that accompany the implementation of ML systems.

As a key epistemic and normative issue, a dispute developed that actually goes far beyond the implementation of ML systems in clinical contexts: It concerns the question of the normative necessity of insightful or instructive modes of generating and presenting ML outcomes. On the one hand, an increasing number of authors emphasize that users need to be able to understand in some way *why* an algorithm delivers a certain result (Robbins 2019) in order not to disappoint the patients’ trust in resulting decisions, and therefore demand from ML-based applications ‘explicable’ or ‘explainable’ generation processes and/or outcomes (cf. Floridi et al. 2018; Holzinger et al. 2020; Rudin and Radin 2019; Heinrichs and Eickhoff 2020; Coeckelbergh 2020; Bjerring and Busch 2021; Funer 2022). On the other hand, some authors consider such ‘explainability’ or ‘explicability’ as an overvalued goal for ML in healthcare, and therefore call for merely demonstrating a certain degree of ‘accuracy’ or ‘reliability’ of the ML system under discussion to sufficiently justify its use (London 2019; Durán and Jongsma 2021). Finally, London (2019) argues, interventions whose underlying causal mechanisms we do not know represent the rule rather than the exception in modern medicine, citing examples such as drug treatment with aspirin or lithium, whose mechanisms of action we have known only rudimentarily for a long time in the first case or even to this day in the second (Ibid.).

One way to resolve these apparently fundamental oppositions seems to lie in a deeper examination of the epistemic and normative foundations of the physician-patient relationship^1^ and in a differentiation of the medical decisions based on these foundations. Through an examination of the epistemic foundations of medical expertise, this paper aims to show why, although empirical ‘accuracy’ or ‘reliability’ is often sufficient, a commitment to transparency for some–perhaps most–medical decisions is nonetheless normatively crucial.

To achieve this goal, I will proceed as follows: In a first step, starting from an example from the recent everyday practice, I aim to highlight what kind of epistemic relationship exists between physicians and patients (2.). In doing so, I will briefly outline some relevant properties of medical knowledge, as well as the privileged access to this knowledge that is usually assumed for physicians. The social-epistemic concept of ‘epistemic authority’ will be helpful. Subsequently, central properties of ML outcomes will be briefly determined, both in terms of their similarities to medical knowledge and in terms of their differences (3.). After examining the two types of information (‘medical knowledge’ and ‘ML outcomes’), I will problematize what challenges might exist when outcomes obtained in different ways contradict each other, and what this might mean for the epistemic role of the physician (4.). In particular, the communication underlying and justifying our interactions (in healthcare) may be jeopardized by the epistemic inaccessibility of ‘opaque’ ML-generated recommendations/outcomes. Therefore, in a final step, crucial aspects for the implementation will be bundled, considering some different decisions to be made in healthcare (5.). A short conclusion summarizes the relevant findings and provides perspectives on the implementation of ML-based systems in the medical practice (6.).

## Medical Knowledge and Epistemic Authority in the Physician-Patient Relationship

I want to start with an illustrating example. Every day in 2021, as more and more vaccines became available, thousands upon thousands of people flocked to vaccination centers and doctors’ offices to protect themselves and others from the SARS-CoV-2 virus, working together to overcome the COVID-19 pandemic. Rarely has our generation been able to participate in the process of researching, testing, and using a vaccine in such an impressive and widely traceable way, with the daily news providing insight into a process that until this time was largely unknown and uninteresting to most people. As the use of vaccines progressed, new insights were gradually gained; not least, information about extremely rare, though sometimes fatal, side effects (e.g., sinus vein thrombosis, capillary leak syndrome, etc.) caused concerns among those potentially willing to be vaccinated. What many researchers took for granted–that empirical knowledge about a new medical intervention is constantly expanding and that this can lead to reassessments, even complete reversals of recommendations–often led to uncertainty and skepticism among those people to whom this process until then had been alien. This manifested itself, on the one hand, in explicit wishes regarding the vaccine to be used and–especially where these wishes proved to be futile–on the other hand, in an increased need for information and education. In this way, physicians attempted to counter uncertainty and skepticism and, where desired, to embed the diffuse information from newspapers, television, and social media in a medical knowledge context and validate its significance for the patient’s specific individual case: With the physician’s help, current information was to be distinguished from information that was no longer current. Information that was considered ‘true’ according to medical knowledge was to be separated from information that was considered ‘false’. And finally, the possible benefits and risks for the individual case should be pointed out and made as comprehensible as possible to enable the patient to make his own decision.

This brief introduction should help us to better understand the communication and interaction between physicians and patients and to identify some (social-)epistemic and normative  dimensions of it. The example I have described is perhaps specific in terms of the subject matter, but paradigmatic for healthcare in terms of the communicative procedure: Patients turn to physicians to obtain, on the one hand, an expert opinion on a specific medical issue and, on the other hand,–thanks to her expertise,–one or more recommendations or alternative options that are considered from a medical point of view ‘reasonable’, ‘valuable’ or ‘advisable’ for the specific individual case of the patient. Consider a little more closely the roles a physician is expected to fulfill in the context of such communication.

### The Physician as Epistemically Privileged Expert (albeit of Uncertain Medical Knowledge)

Let us first take a closer look at the aspect of an assumed medical expertise of the physician: What is a more precise (social-)epistemic understanding of the relationship between physicians and patients? As beings with limited capacities, each of us depends on other persons, which in societies with a highly differentiated division of labor often leads to epistemically asymmetric relationships or dependencies (Martini 2020; Fricker 2006). In the face of one’s own inability in a subject matter, it turns out to be efficient to consult those persons who have more extensive knowledge and higher competencies in this specific domain. For the matter of health and medicine, physicians, thanks to their training and experience, can be described as professional experts. Insofar, vis-à-vis their patients, which are–sometimes more, sometimes less–medical laypersons, physicians act as domain-relative ‘epistemic authorities’. The patient relies on the epistemic authority of the physician and trusts her epistemically, that is, believes in the truthfulness of the physicians’ propositions thanks to her epistemic authority on the matter (Keren 2007).

Goldman (2018) describes such expertise as follows: “S is an expert in domain D if and only if S has the capacity to help others (especially laypersons) solve a variety of problems in D or execute an assortment of tasks in D which the latter would not be able to solve or execute on their own. S can provide such help by imparting to the layperson (or other client) her distinctive knowledge or skills.” There is now a broad discussion about the extent to which expertise depends on propositions or beliefs which are actually true (Goldman 2001; Keren 2007) or with which properties epistemic expertise correlates (Martini 2020). However, numerous internal and external factors are relevant to designate a person as an expert, such as objectivity, unbiasedness, content- and meta-knowledge, etc. (cfr. Martini 2020). And these factors can, if necessary, be subjected dialogically to arbitrary (re-)evaluation in social interaction between expert and layperson. In this respect, the epistemic expertise of the physician, though mostly inaccessible to the layperson in terms of content, remains at least communicatively interrogable in terms of its general rational criteria (evidence, reasonability, consistency, deliberation). Goldman therefore understands expertise in a broader sense as a productive and systematic capacity: „Expertise is not at all a matter of possessing accurate information. It includes a capacity or disposition to deploy or exploit this fund of information to form beliefs in true answers to new questions that may be posed in the domain. This arises from some set of skills or techniques that constitute part of what it is to be an expert” (Goldman 2001).

I want to direct the focus of the argument here to the expertise of physicians as a productive and systematic capacity. This particularly because of the nature of medical knowledge: large parts of our medical knowledge are first based on empirically collected and statistically analyzed data, that is, are evidence-based. From different forms of statistics, probabilities are collected for certain predictors, constellations, progressions, endpoints, etc. In this way, our medical knowledge probabilistically comes closer and closer to reality ‘as it really is’ (cf. for approximate truth e. g. Putnam 1982; Hardin and Rosenberg 1982; Smith 1998), with the aim of representing it as best as possible. Nevertheless, there is inevitably an, what I call, ‘epistemic gap’ between this set of information, which is based, for example, on generalizations, categorizations, and cancellations of so-called ‘statistical outliers’, and the concrete individual case at hand with its unique circumstances. The individual patient embodies reality as it really is and is not a statistical representation of it. The patient therefore eludes statistical simplifications, cannot always be captured in categories, or may just *be* a statistical outlier. Categorically, statistical findings, even if taken all together, cannot describe the reality exhaustively because of their simplifying basic design (cf. the problem of external validity in Solomon 2015: 141 ff.; Worrall 2007). Of course, the physician cannot consider all probabilistic outcomes of evidence. Instead, she relies on rationalization attempts and theorizations of the scientific community to obtain a picture of the area of her expertise as complete as possible. To describe such domain-specific understanding, Catherine Elgin used the notion of a reflective equilibrium within one’s thought system (Elgin 2017, pp. 3–4), which “consists not only of particular factual judgements or beliefs, but also of theoretical commitments, acceptances, generalizations, methods of inquiry, standards of justification, and epistemic goals and values” (Jäger and Malfatti 2020). In this way the physician succeeds, even if only rationally, in creating a ‘noetic network’ that can close, or at least narrow, the epistemic gap between empirical probability generalizations and the concrete individual case at hand. In a nutshell: To address the individual situation sufficiently, the application of statistical findings into clinical decision-making requires “a good deal of background knowledge” (Solomon 2015: 141 ff.; cf. Cartwright 2007a/2007b).

The treatment of an individual patient therefore requires an interpretative capacity that examines, evaluates, and selects the existing medical knowledge regarding to its relevance for the concrete individual case. For this, the information must be integrated into a network or ‘order’ of the other epistemically well-founded propositions or beliefs. In doing so, the physician can serve, with her professional recommendations and judgments, as a “source of learning” (Jäger and Malfatti 2020) for the patient’s decision.

Let us illustrate this within the introductory example: The patient who is willing to be vaccinated, who has heard a lot about the risks and chances of the available vaccines, comes to the physician to be professionally informed by her about the current state of knowledge to make his own *informed consent*. The physician will–probably–first provide information about the current empirical situation, the probabilities of achieving effective vaccine protection, the possibility of non-responding, and the likelihood of expectable vaccine reactions and adverse effects or complications. While some patients will already be satisfied with this information for their decision-making, probably some of them will still feel insufficiently informed. Perhaps they want to be able to interpret the empirically determined probabilities for their individual situation to make what they believe to be a self-determined decision. The physician is now challenged to approximate the case of the individual patient in a factually adequate way on basis of the different data and rationalization attempts. She will do this as extensively as she is capable and willing to take (professional) responsibility for her recommendation, based on her epistemic convictions or beliefs–always under the potential obligation to justify them in a deliberative process with the patient or in front of third (cf. Coeckelbergh 2020).^2^ Thus, the assumption of professional responsibility requires at least the professional, formed as an expert, conviction of the proposition at issue; a fortiori, to communicate it to the patient for his decision-making.

### The Physician as Morally and Normatively Trustful Adviser (thanks to her Epistemic Authority)

Let’s take a brief look at the second function that a physician usually has to perform in the context of communication with the patient (cf. e.g., Mallia 2013: 29 f.), and which is based on the epistemic relationship described up to this point: The physician is called upon to present possible alternative courses of action, that is, treatment options and individual measures, to the patient for his concrete individual case, and to evaluate these together with the patient in the sense of shared decision-making. Although the task to morally evaluate possible alternative courses of action is ultimately incumbent on the patient himself, he is nevertheless dependent for this purpose on the epistemic expertise of the physician. The patient is originally best informed about his biographical, situational, personality-related, social, religious, and moral dimensions of life, and therefore best aware of his own interests, values, and goals. But in most cases, he cannot apply all of them without any substantive information and experience of the physician.

For this reason, within the deliberative process with the patient, the physician will not be allowed to present only the empirical ‘hard facts.’ Furthermore, she will have to empower the patient undertaking mediations between the ‘medical knowledge’ (cf. Solomon 2015) and the patient’s interests, values and goals. In this kind of relationship, which Emanuel & Emanuel have designated as the “*deliberative model*”, the physician “must delineate information on the patient’s clinical situation and then help elucidate the types of values embodied in the available options” as well as to suggest “why certain health-related values are more worthy and should be aspired” (Emanuel and Emanuel 1992). Finally, she will have to point out to the patient the possible limitations, uncertainties, and ambivalences of the medical knowledge regarding the situation. The fundamentally different heuristics persons are using for health-related decisions make medicine a hermeneutic activity, an “interpretive meeting between health-care personnel and patient with the aim of healing the ill person seeking help” (Svenaeus 2001: 2; cf. also Svenaeus 2018; Neighbour 2016: 152 f.; Mallia 2013: 71 ff.; more in Sect. 4).

This epistemically demanding capacity on side of the physician requires an enabling exchange, in which the patient as decision-maker, even if he does not know the details of the content, should at least have the opportunity for a communicative assessment of general criteria of epistemic expertise (evidence, reasonability, consistency, deliberation; cf. Martini 2020) and of their moral and normative implications (more in Sect. 4). Only in this way does the epistemic expertise of the physician allow her to present this or that alternative course of action as preferable and therefore more recommendable in a normatively justified manner–that is, in a rationally justified and thus for the patient deliberatively accessible manner–in view of the patient’s own interests, values and goals. It is true that the epistemic expertise of the physician *enables* her to give advice to the patient; but as long as we hold on to some sort of informed or autonomous decision-making by the patient, only the (potential) deliberative accessibility of the reasoning *legitimizes* the physician’s giving of advice.

Let us return to our vaccination example: The physician has now presented the potential advantages and disadvantages to the patient from an evidence-based perspective and provided them with probability values. But to evaluate morally these probabilities for her own situation the patient requires more “epistemic empowerment.” However, the physician will be able to help the patient in assessing and evaluating potential risks if and only if she explains to him how the probability values came about, offers him possible attempts of plausibilization, and can explain to him why she, as epistemic authority, comes to her epistemic belief or judgement regarding the possible vaccination in the individual case in question. Even if by no means all patients demand, let alone desire, such a deliberatively elaborated and autonomy-respecting interaction, the potential capability and professional normative obligation of the physician to provide reasons for her belief or judgement establishes the patient’s trust in her as epistemic authority.

But what does this apparent digression into the epistemically and normatively deliberative relationship between physicians and patients teach us about the use of ML technologies? As will become apparent, the implementation of ML applications in this relational and communicational structure is by no means a task to be undertaken lightly or reckless.

## ML-Generated (Medical) Outcomes: Is there Something New About it?

Recent developments in the healthcare field owe primarily to significant advances in data generation and processing, as well as breakthroughs in ML. Here non-rule-based algorithms ‘learn’, that is, generate insights, by recognizing certain patterns and regularities in a defined set of raw data (Hinton 2007; Schmidt-Erfurth et al. 2018; Ahuja 2019). The goal of ML, sketched roughly, is to ‘intelligently’ link data, identify relationships, draw conclusions, and make predictions–and this without being explicitly programmed in its entirety. The complex architecture of ML algorithms, especially those using deep neural networks, makes it difficult or even impossible to understand how variables are combined to make such predictions or recommendations (cf. Zednik 2021; Rudin and Radin 2019). Basically, “deep neural networks consist of layers of nodes that each use simple mathematical operations to perform a specific operation on the activation of the layer before, leading to the emergence of increasingly abstract representations of the input image” or other data (Grote and Berens 2020). For these multi-layered networks can be hold, the larger the underlying data set, the better the outcome produced by the algorithm (Hinton 2007; Ahuja 2019). With the accumulation of input instances, the relative weights of the various nodes in the neural network adjust themselves to achieve the most accurate mathematical representation possible of the fed-in state of information. Thanks to classifications tested on extensive data, ML enables highly accurate statements about probabilities of the presence of a certain finding (e.g., diagnostic image analysis) or the occurrence of a certain event (e.g., prognostic chances of therapeutic success/failure). Since the algorithm is fed by collections of overwhelmingly large amounts of data, its development is “neither foreseeable nor transparent to the programmer” (Heinrichs, 2020). London (2019) illustrates this phenomenon of ‘black boxes’ as follows:

Even when techniques are used to identify features or a set of features to which a model gives significant weight in evaluating a particular case, the relationships between those features and the output classification can be both indirect and fragile. A small permutation in a seemingly unrelated aspect of the data can result in a significantly different weighting of features. Moreover, different initial settings can result in the construction of different models.

Such systems are therefore mostly characterized by a certain degree of ‘epistemic opacity’. In this context, epistemic opacity means that the complex and multi-dimensional mathematical processing performance of the algorithm so far is not (fully) comprehensible by means of the understanding and language of human agents. Yet, while for some processes forms of explicable or even interpretable^3^ ML systems can be developed^4^, other very complex procedures that take immense amounts of data into account may never be explicable to human agents due to their limited processing capacity. Of course, some systems may be more accessible to informatics experts than for example to a doctor or a patient, which is why we can also speak of a “relative concept” (Smith 2021). But if a system is described as epistemically opaque, it evades sufficient interpretation and remains to some degree inaccessible to everyone.

Now, London (2019) attempts to clarify in his remarks that this circumstances of opaque, that is, not interpretable, outcomes by ML are not a novelty in healthcare, drawing parallels between the opacity of ML-generated outcomes and judgments formed “in the clinician’s head that is opaque and often inaccessible to others” (Ibid.). According to him, this is possible because the “explanatory power” of *all* medical knowledge is questionable (London 2019): While in other fields a comparatively high completeness of causally significant interrelations has been achieved or at least can be achieved, according to him the knowledge about underlying causal interrelations in medicine is said to be merely “in its infancy” (Ibid.). Pathomechanisms as well as therapeutic modes of functioning are often unknown or poorly understood, he said, for which reason “decisions that are atheoretic, associationist, and opaque are commonplace in medicine” (Ibid.). Large parts of the empirical knowledge in medicine were applied for many years, although there was or is no causal insight into their mechanism of action, as in the case of aspirin or lithium (Ibid.). Other therapies that were based on causal hypotheses–that is, theoretical attempts of explanation–ex post had turned out to be wrong. Rigorously gathered empirical findings are therefore said to be more reliable and to *reflect* causal interrelations better than “theoretical claims that purport to ground and explain them” (Ibid.). Our medical knowledge and practice, he concludes, would be mostly “a mixture of empirical findings and inherited clinical culture”, why recommendations based on it “reflect experience of benefit without enough knowledge of the underlying causal system to explain how the benefits are brought about” (Ibid.). Without being able to present exhaustively London’s illustration of his point here, I would summarize his argument roughly as follows: Since uncertainty, especially one involving causal interrelations, is the rule rather than an exception in medical practice, and clinical decisions therefore often originate in the physician’s apparently opaque and often inaccessible neural network, equally opaque recommendations of artificial neural networks are “not radically different” from it. And consequently, while our ability to consider numerous features remains fragmentary and thus limited, non-rule-based algorithms are comparatively superior to us in terms of their ‘accuracy’ and therefore sometimes preferable. Hence, the focus of our attention should be less on efforts in terms of explicability or interpretability of ML-generated outcomes, but rather on the empirical validation of their accuracy (Ibid.).

## Misunderstandings about the Epistemic and Normative Value of Physician-Patient Communication

Although the parallels London (2019) points out may enjoy intuitive plausibility, I think they are misguided with respect to at least the following two (social-)epistemic and normative properties of the deliberative physician-patient relationship, and thus fail to recognize the difference to ML-generated outcomes:

Firstly, I disagree with the *kind of the physician’s opacity*. Recommendation or decision-making in medical practice unfortunately may sometimes appear quite ‘opaque’ to patients. But nevertheless, in the case of human agents, who make decisions and perform actions, there is basically the possibility of questioning the physician about the epistemic and normative foundation on which she has built her recommendation or decision, of comprehending the rationale of her recommendation or decision as far as possible, and of checking its plausibility regarding general criteria of epistemic expertise (cf. Section 2.1; also, Martini 2020). The deliberative process enables the patient to interrogate the physician for her justification(s), to evaluate her epistemic expertise (at least in part)^5^ as well as her plausibilization attempts for making her recommendation or decision. In this way the patient will determine her as *epistemically (not) trustworthy*. Opaque ML outcomes, on the other hand, are characterized precisely by the fact that they do not possess such basic possibility of inquiring into their epistemic justifications. With their ‘accuracy’ or ‘reliability’ a high degree of epistemic expertise or ‘certainty’ is *claimed*, which, however, categorically resists rational scrutiny and communicative deliberation due to its opacity. Instead of being able to incorporate the outcome into one’s own epistemic order, the agent–the physician as well as the patient–is left with relatively little information.

Secondly, I question the *negligence of moral content and normativity in most medical decision-making.* London’s point that medical recommendations and decisions often lack sufficient insight into the underlying causal interrelations does not seem to me to be fundamentally wrong with respect to medical knowledge in a narrow sense, that is, one that equates medical knowledge mostly with encyclopedic and up-to-date evidence-based knowledge. But that is not all. Neighbour (2016), for example, criticized such a one-dimensional conception under the term of a “conventional medical model”, as it were, an oversimplified application of Occam’s razor^6^ in medicine, according to which disease is thought as a (patho-)physiological straightforward linear sequence of cause and effect (cf. ibid.: 113). Encouraged by the hopes of pioneers of evidence-based medicine, in which all diagnostic and therapeutic questions would be answered based on incontrovertible evidence and applied in a systematic Bayesian way to every clinical dilemma (cf. ibid.: 190), similar hopes are now being applied to ML-supported healthcare.

However, most decisions to be made clinically involve much more than only (patho-)physiological mechanisms or pharmacological modes of action, so involve more than only a ‘medical point of view’. Of course, this is by no means something new in medicine and healthcare (cf. for this e.g., Wiesing 1995). The epistemic problem underlying was elaborated by Solomon (2015), namely that medical knowledge is not only one form but is characterized by a *variety of methods and heuristics* (e.g., ‘consensus conferences’, ‘evidence-based medicine’, ‘translational medicine’, and ‘narrative medicine’). Due to its property to provide perhaps more than one appropriate method to describe and address the same problem, different results and thus incoherence with different pursuable goals in treatment may occur. And to make this clear: This does not mean that the different pursuable goals vary only in terms of their probability of addressing the identified disease in an adequate manner.

In fact, many clinical recommendation and decision-making processes have to take into account implications whose depth of intervention or scope for the patient’s life and its quality goes far beyond the one-time intake of a medication that is not fully understood yet.^7^ Even research on the implementation of ML recommendations today involves normatively far weightier therapeutic decisions, for example, on the admission and (possibly over long periods) continuation of treatments that severely restrict or potentially endanger the patient’s life (cf. ‘Watson for Oncology’); decisions whether a patient is considered capable of giving consent; decisions on the continuation of life-sustaining measures or resuscitation. Such decisions concern numerous other dimensions beyond the purely evidence-based findings and operationalizable characteristics of a person. The selection of parameters deemed relevant, the weighting of each possible treatment goal, the choice of means to achieve the selected goals with their respective consequences for the patient’s life, these and many other aspects are inherently normatively charged. Therefore, the pursuable goals in treatment vary in terms of their moral value and normative weight depending on how the patient views his disease, life, and personal situation, and which goals–beyond the pathophysiological describable deficits–he considers valuable or significant to pursue within treatment (cf. Neighbour 2016: 179).

The deliberative practice between physicians and patients makes it possible to obtain at least an impression of the respective other understanding of terms like health, disease, and suffering, to determine moral and normative implications at different levels of shared decision-making, and thus, in an ideal-typical manner, to ‘sharpen’ individually the picture of the patient’s interests, values, and goals regarding the situation at hand (cf. also Funer 2022; Mallia 2013).^8^ Even mismatches or misunderstandings can be identified and circumvented (Kiener 2021). This enriched picture subsequently helps in the evaluation, hierarchization and selection of possible evidence-based diagnostic and therapeutic alternatives. Svenaeus (2018: 62–69) elaborated similarly his method of ‘medical hermeneutics’ with recourse to Hans-Georg Gadamer. Regarding to him, the aim is to “bring together the horizons” of the physician and the patient. In dialogue, the two different ‘horizons of understanding’ were “aimed at establishing a mutual understanding which can benefit the health of the ill party” (Ibid.: 65; cf. Sveneaus, 2001). Physicians, he concludes phenomenologically, are “thus not first and foremost scientists who apply biological knowledge but, rather, interpreters–hermeneuts of health and illness” (Svenaeus 2018: 65; cf. also Mallia 2013: 71 ff.).^9^ Even Mallia (2013: 73) concludes on basis of the Gadamerian hermeneutics: “All interpretation is grounded on understanding. In so far as judgements and assertions are grounded on understanding and present us with a derivative form in which an interpretation has been carried out, it too has ‘meaning’.”^10^ In this way, decisions can be made that link the existing empirical medical knowledge with patients’ individual evaluative judgments. The consideration of this enriched picture of the patient, verifiable in a relational-deliberative way, is significant for him to determine the physician also as *morally and normatively (not) trustworthy*.

If an opaque ML system does not make its integrated moral and normative implications transparent, it evades such deliberative scrutiny and makes adequate evaluation by the physician and the patient difficult or even impossible. Now, some may believe the epistemic authority of the physician is as such groundless or invalid if the ML system achieves a higher accuracy or reliability within its outcomes. However, they fail to realize that this also deprives the physician of his justification, namely qua his epistemic expertise in the question, to give moral and normative recommendations to the patient. The importance of an appropriate implementation of ML in our relational-deliberative practice will be illustrated in the following section.

## “Peer”-Disagreement in Case of Epistemic Incompatibility–A deliberative Worst-Case-Scenario

Of epistemic and normative explosiveness could be those situations in which the outcome of an ML system contradicts that one obtained in a ‘conventional way’ (i.e., by means of established instruments and based on existing medical knowledge) and–possibly due to a lack of insight–also cannot be validated. How likely such situations are, which in social epistemology are often discussed as “novice/two-expert problems” (Goldman 2001), remains questionable. Nevertheless, such situations represent a possibility that must necessarily be considered, because it confronts the physician with an epistemic and normative challenge: For Grote and Berens (2020), “peer-disagreement” describes “cases of two (equally) competent peers with respect to a certain domain-related activity, whereby both parties disagree with respect to a certain proposition” (Ibid.). So, in our case, we would be dealing with a situation where, on the one hand, a physician would act as a medical expert thanks to her training and experience, and on the other hand, an ML system would seem to act as a medical expert thanks to its enormous datasets and categorizations. Both would disagree on a certain proposition, e.g., a clinical recommendation to be made. The authors therefore ask: “Now, when trying to make a well-informed decision, how much weight should the clinician assign to the algorithm’s diagnosis? Bluntly put, should she be required to call her superior out of bed for an additional opinion? Or, would the superior be rightfully mad, given that the algorithm provided a clear diagnosis?”, and rightly summarize, “[t]here is very little that the clinician might do on epistemic grounds to resolve the disagreement in question” (Ibid.). Differing, sometimes even contradictory expert opinions are, of course, not uncommon in medical practice either. They may arise due to the inherent ambiguity of clinical symptoms and disease phenomena and their perception by different experts (Cabitza et al. 2017), as well as due to variable individual or disciplinary weightings considering the achievement of a particular goal (lifetime, mobility, independence, quality of life, etc.). In fact, medical practice is genuinely characterized by uncertainty and ambiguity (cf. Neighbour 2016: 183 ff.). However, whereas two human agents who arrive at divergent beliefs regarding a proposition can, at least in principle, be questioned about their epistemic justification of their expertise proposition (Martini 2020), this is not the case with opaque ML systems. Based on these characteristics, at least two conceivable constellations arise for practice, but neither can solve the problem of epistemically incompatible outcomes: an *ML-supported physician-patient relationship* or some kind of *triangulated physician-patient-ML relationship*.

In the first case, the physician would have to face the epistemic challenge: But the physician may lack the tools or the medical knowledge to both verify or falsify the outcome generated by the opaque ML system. First of all, such a conceivable constellation offers surely potentials of treatment improvement, since the physician inevitably has to re-examine and re-evaluate the measure, she has favored so far, in order to exclude possible errors. However, if she still comes to the conclusion that the measure she recommends continues to deviate from that one of the ML system, she would have to decide whether to maintain her belief despite the divergent outcome, or to adjust it, or to abandon it (Grote and Berens 2020).^11^ Yet, she could not do so based on reasons because the opaque ML outcome does not disclose its reasons–it is an epistemic stalemate situation in which only different justification strategies can be invoked for the divergent results (different kind of reasons on the one side vs. high accuracy/reliability on the other). By adopting the outcome that cannot be rationally integrated because of its opacity, the physician loses her epistemic authority regarding the certain proposition. She does not distinguish herself by an increased expertise about it since her complementary knowledge and experience cannot help her in its interpretation. Due to this inaccessibility and subsequently impossible interpretation or integration into her own epistemic order, the physician in any case could not bear epistemic and normative responsibility for the recommendation generated by the epistemically opaque ML system (Smith 2021).

Even the second form, the triangular relationship, in which the algorithm would have its own epistemic and normative status and which would counter insofar the lack of accountability by the physician seems to be ineligible, because choosing between two epistemically incompatible ‘paths of conviction’ cannot be solved by the patient either. Putting the responsibility for an epistemically unsolvable question over to the patient–especially in her vulnerable situation–is doubtful.

It becomes evident that the embedding of an ML outcome into the deliberative and communicative recommendation- and decision-making process would clearly benefit from a maximum possible insight into the genesis and thus interpretability of this outcome. Therefore, I propose for the implementation of ML-generated outcomes, the more normatively far-reaching and important the clinical decision to be made and so the higher the justification requirements applied to this decision, the more significant are traceable and interpretable processes of the ML-based generation of recommendations to be considered for this decision.

## Implementing ML Support in the Deliberative Physician-Patient Relationship

The advantage of interpretable systems is that the agent using them, e.g., the treating physician, optimally obtains an insight into the adequate or perhaps non-adequate factors considered^12^, into the fitting of the data clusters used to the concrete individual case at hand, into perhaps unilaterally translated ambiguities into ML-appropriate operationalizations, into the weightings made and, if necessary, into the dimensions of the patient’s personal life unconsidered so far by the algorithm. Certainly, this represents an extensive requirement for transparency (cf. Funer 2022). But only in this way it empowers the physician and the patient to undertake their plausibility attempts for their own judgement^13^, that is, to integrate the ML-generated information and recommendation into their own already existing order of knowledge and experience–or to reject it(!).

ML-generated information, which cannot or can hardly be integrated into this order due to its lack of transparency, can therefore be problematic: That to which one person (e.g., the physician) has no intelligible access, she cannot interpret–integrate into her own epistemic order–and therefore cannot epistemically and normatively evaluate for herself, much less convey to another person (e.g., the patient) for her own evaluation. However, these interpretative and evaluative aspects represent central facets of the communicative physician-patient relationship with its division of responsibility.

Physicians and patients succeed in their shared decision-making because of the epistemic and normative trust they have in each other. Indeed, this is not ‘blind trust’ as long as both sides have the possibility to deliberatively examine their trust regarding its epistemic foundations and normative implications. To trust another person is to grant her epistemically and normatively such authority, hence, to recognize her as issuing a preemptive reason to believe, what she is saying (cf. Keren, 2020). But only persons who *respond to reasons* can issue such preemptive reasons for their propositions and therefore deliver trustworthy judgements (Ibid.). ML recommendations that are not adequately accessible for the situation at hand, that is, that are not responsive to reasons, would hinder such a possibility for scrutiny and reduce the relational foundations of trust to only technical parameters (such as accuracy/reliability) that claim higher certainty. However, this claim of higher certainty is a fallacy because it is based on reductionisms and simplifications of an intrinsically uncertain and ambiguous medical reality (cf. Cabitza et al. 2017; Bjerring and Busch 2021; Mallia 2016; Wiesing 1995).

The complex interaction between patients and physicians, in which each decision to be made shows different justification requirements, does not preclude ML recommendations, even opaque ones. The challenge of an ML-supported physician-patient relationship now consists in the identification of precisely those decisions that largely do not require normative justification (“medical in the narrow sense”). Such decisions could be supported analogous to evidence-based guidelines–in compliance with defined duties of care and quality standards as well as under exclusion of basal undesirable biases–, which bundle the current empirical state of knowledge and pre-sort it regarding some criteria suitable for the individual patient (age, sex, pre-existing conditions). The treating physician could then further use these outcomes herself, considering the other non-operationalizable but relevant factors, and considering existing ambiguities and uncertainties. For those decisions that require a higher normative justification due to their scope and depth of intervention, forms of interpretability and insight into the genesis of the recommendation will be important for implementing them in the deliberative practice of physicians and patients.

The requirements for (potentially necessary) justification of recommendation- or decision-making, though perhaps only rudimentary or even sometimes retrospectively proven to be wrong, nonetheless form the normative basis of our interaction between people; this even more in the necessarily trust-based physician-patient relationship.

## Conclusions

*“True genius resides in the capacity for evaluation of uncertain*,

*hazardous, and conflicting information.”*

(Winston Churchill, 1874–1965)

As should have become clear, ML-based systems and human agents differ in terms of their assets in the process of making clinical decisions. The former, thanks to their gigantic processing capacities, are potentially capable of data-based synthesis performances that surpass human capacities many times over. The latter, in turn, are potentially capable of a discernment or integration performance by considering all those normatively relevant aspects that go beyond operationalizable data (social-relational, psychological, moral, religious factors) and by perceiving and processing uncertainties and ambiguities that will remain ineligible to ML-based systems for the foreseeable future, due to the lack of existing and perhaps never fully operationalizable data. Both ML systems and human agents have different kinds of error-proneness and weaknesses, which the other seems to be able to improve.

What I have tried to describe is how the epistemic authority of the physician in clinical decision-making situations can be abrogated if non-interpretable ML outcomes were used. Of course, merely this description of a change in the epistemic and normative role of the physician does not constitute a sufficient reason why non-interpretable ML should not be used in healthcare. However, their use does call into question which epistemic and normative role the physician *can* have in shared decision-making at all, and therefore requires intensive reflection on the requirements of our so far valued health care maxims of informed consent and patient autonomy.

The task for the future in the development and implementation of ML systems in healthcare will therefore be to identify that equilibrium in which the skills of physicians and ML systems complement each other in the best possible way. The communicative requirements of the respective decision-making situation are of decisive importance here. To achieve this, ML developers and physicians will not be able to avoid close collaborations. While on the one hand regulatory quality standards and performance criteria for the evaluation of the achievement of medical benefits and of the compliance with other relevant aspects (privacy, liability, etc.) have to be elaborated, on the other hand it is necessary to search for and formulate as precisely as possible implementation opportunities in clinical practice: Only by taking into account the concrete potential decision-making situation, those aspects and goal perspectives of the decision can be identified whose outsourcing to the ML system is responsibly possible, or which must necessarily remain comprehensible for the clinical decision-maker and thus assessable and communicable to the patient. In the process of clinical decision-making, this will help both the physician and the patient to assess, avoid or consciously accept possible risks.

## Data Availability

Not applicable.

## Footnotes

1 The term ‘physician-patient relationship’ is used here as more common in the clinical jargon, but it can equally be applied for relationships between patients and other health-care professionals.

2 Coeckelbergh (2020) similarly analyzes “responsibility as answerability”. On the one hand, to be responsible *for** something*, the agent must know what she is responsible for. Knowledge enables her to exercise responsible agency. But on the other hand, responsibility is also about being responsible *for someone*, that is, a person who is affected by the action of the agent: “Responsibility is not only about doing something and knowing what you’re doing; it also means answerability. It is also a relational and communicative, perhaps even dialogical matter” (Ibid.).

3 I do not necessarily want to defend the term ‘interpretability’ here. Krishnan (2020), for instance, pointed out that this term often masks other ends pursued, such as justification or non-discrimination. In fact, I am mainly concerned here with epistemic and normative justification. I refer to interpretable information as one that can be integrated by the agent into her own order of existing knowledge.

4 So far, two possible approaches exist (Heinrichs and Eickhoff 2020; Hutson 2021; Molnar 2021): On the one hand the *explicability of the operating mode of an entire algorithm* (‘global’ or ‘model explicability’). Here, the goal is to give the interacting user the best possible insight into how the algorithm works in general. Such global explicability is usually achieved by using interpretable ML (iML) systems to approximate the predictions of ‘black box ML’. By interpreting the iML, we can then draw conclusions about the black box model itself (cf. Molnar 2021). On the other hand, the *explicability of certain singular outcomes* (‘local’ or ‘outcome explicability’) focuses on the features selected and weighted in the specific individual case, which may be of particular interest to the physician and the patient. To convert singular opaque outcomes into interpretable ones, local surrogate models, such as Local Interpretable Model-agnostic Explanations (= LIME; cf. Molnar 2021; Visani et al. 2020), are used today. LIME approximates, through numerous tests on the opaque system, what happens to the individual outcome when the underlying data set fed into the black box is changed. For a more detailed overview of currently possible explanatory methods and instruments cf. Molnar 2021.

5 Perhaps one might now object that patients are mostly unable to judge the expertise at all. Nevertheless, I consider the possibility of being able to ask the physician about general criteria of how her recommendation or decision came about, or potentially to consult someone else–with supposedly greater expertise in the field–to be achievements of the deliberative relationship between physicians and patients. The fact that this is not always realized by the patient does not in any way mean that the possibility of doing so is irrelevant.

6 Occam’s razor is a heuristic principle according to which the greatest possible parsimony should be applied in explaining a hypothesis or phenomenon, and therefore as few assumptions as possible should be made (cf. Neighbour 2016: 113 f.).

7 Of course, Alex J. London is to be agreed that we are perfectly content with the demonstrated accuracy in the context of the therapeutic use of, say, medications such as aspirin or lithium, even if we lacked, at times, or even lack now, explanations, even probabilistic certainties, about their functioning (London 2019). However, the depth of intervention and the scope of the decision to take a medical preparation (once) is, excluding the most important contraindications, exceedingly manageable and can also be compensated to a large extent afterwards.

8 In this sense, it is correct to say that statistics about disease progressions, average survival times, recoveries, and deaths cannot afford such an enriched picture of a patient. To what extent other aspects mentioned could be operationalized at all–and thus be sufficiently considered in the future–,I am not able to judge.

9 In his phenomenological approach, Svenaeus (2018: 74) uses the notion of “empathic understanding” that would develop into “interpretative understanding” of the other person’s being-in-the-world. The entire medical understanding, regarding to him, is therefore “richer than and different from explanations of bodily dysfunctions only, since it is about a person and her life world, about the way she embodies core life values by way of a narrative” (Ibid.). Interesting here is also the effect worked out by Neighbour (2016) that what he calls the “inner doctor” (i.e., his personality and biography or his being “expert minus the expertise”; ibid.: 249) can achieve in the dialogue with the patient.

10 For more cf. Mallia (2013) and Svenaeus (2001/2018).

11 Grote and Berens (2020) made it clear that it is still nearly compelling for a physician, especially a professional ‘novice’, given the correctness suggested by the system design (e.g., that ML algorithms have been trained based on data from numerous other experts), to defer to the ML outcome.

12 Some of the numerous potential biases have been sufficiently pointed out elsewhere (London 2019; Hutson 2021). Nevertheless, such biases are not a novelty in medical knowledge generation. Consider for example the long known but nevertheless insufficiently considered appeals of ‘gender medicine’ that physiological differences between the sexes should lead to different therapies (Baggio et al. 2013). It is obvious that our medical knowledge is also biased with respect to other factors, such as ethnicity and the like. However, if biases remain undetected, they cannot be taken into account in the interpretation and application in individual cases.

13 Individual variety in the assessment of uncertainties and ambiguities in front of one’s own life was also pointed out by Neighbour (2016: 178 f.): “Someone else might have had a different tipping point, or have weighted the evidence differently, or have differently assessed the relative dangers of action and inaction. Politicians and doctors are equally familiar with the difficulty of having to base their decisions on unreliable information. A dozen times a day the practising clinician wonders, ‘Given this degree of uncertainty, what is the best thing to do?’ … So–since imprecision, uncertainty and ambiguity are givens in most clinical situations–alternative diagnostic strategies have to be found to bring doctor and patient to ‘the verge of action’. The linear rationality of the medical model, while useful in a crisis, is not well suited to unpacking the fuzzy complexities of many workaday consultations.”
